# Computational studies on Begomoviral AC2/C2 proteins

**DOI:** 10.6026/97320630014294

**Published:** 2018-06-30

**Authors:** Kanthalu Shagadevan Dinesh Babu, Prabu Manoharan, Gopal Pandi

**Affiliations:** 1 Department of Plant Biotechnology, School of Biotechnology, Madurai Kamaraj University, Madurai, India; 2 Center of Excellence in Bioinformatics, School of Biotechnology, Madurai Kamaraj University, Madurai, India; 3 Department of Biotechnology (DDE), Madurai Kamaraj University, Madurai, India

**Keywords:** Gemini virus, AC2 protein, Transcriptional activator protein, acidic activation domain, protein interaction, CLE elements, Host- gene transactivation

## Abstract

Geminiviridae is a large family of circular, single stranded DNA viruses, which infects and causes devastating diseases on economically important crops. They are subdivided into nine genera. Members of the genus begomovirus encode a pathogenic protein called AC2/C2 which interacts that inactivates many plant proteins and trans-activates a number of host genes via the C-terminal transactivation domain. Hence, a sequence analysis on C-terminal region of AC2/C2 was completed. Analysis of 124 bipartite and 463 mono partite begomo viral AC2/C2 proteins revealed major differences in protein length, composition and position of acidic, aromatic and hydrophobic residues. Secondary structure analysis of AC2/C2 revealed the possible formation of C-terminal α-helix, which is similar to the acidic activation domain of many transcriptional activator proteins. Previous studies demonstrated that AC2 utilizes conserved late element (CLE) for the transactivation of viral genes and genome-wide mapping of same consensus in A. thaliana yielded 122 promoters with exact CLE consensus sequence. Analysis of protein interaction network for 106 CLE containing genes, 87 AC2 trans activated genes and 10 AC2 interacting proteins revealed a possible regulation of hundreds of host proteins which helps begomoviruses to produce a successful viral infection.

## Background

Geminiviridae is a large family of plant infecting viruses, which has characteristic twinned-icosahedral particles. They produce devastating diseases in many economically important crops and cause more than 95% yield loss [1]. They were classified into nine genera based on their genome organization and the type of insect vector involved in their dispersal [2]. Among the geminiviruses, the genus begomovirus is a large group with more than 350 members, transmitted by the whitefly (Bamesiatabasi). Begomoviruses can be either monopartite (having a circular single stranded DNA (ssDNA)) or bipartite (having two equal size circular ssDNA known as DNA A and B) with varying genome size ranging from 2.5 to 5.2 kb. They replicate inside the plant nucleus via rolling circle replication (RCR) [3]. The A DNA encodes 4- 5open reading frames (ORF's) in complementarysense direction (AC1/C1, AC2/C2, AC3/C3, AC4/C4 and AC5/C5) and two ORF's in virion-sense (AV1/V1, AV2/V2) direction which are involved in transactivation, replication and encapsidation [4]. DNA Bencodes nuclear shuttle protein (NSP or BV1), Movement protein (MP or BC1) and both are exclusively involved in viral DNA movement [5]. There is a unique kind of circular ssDNA satellite molecules found associated with the begomo viruses called alpha and beta satellites [1, 6, 7]. The beta satellite encodes only one ORF called βC1, which is essential for typical symptom production and suppression of RNA silencing. Alpha satellite encodes a replication-associated protein (Rep), which allows its autonomous replication inside the plant cell [7].

Upon geminiviral infection, certain viral proteins activate the transcription of virion-sense genes of viral genome and innumerable host cellular genes for making the cellular environment favourable for viral establishment. This phenomenon is known as transcriptional activation and the protein responsible for this process is called transcriptional activator protein (TrAP) [8, 9, 10]. The members of the genus begomovirus encode a small multifunctional AC2/C2 protein which is regarded as a TrAP of viral genes and suppressor of RNA silencing [10, 11]. The multifunctional AC2/C2 does direct interaction with number of host genes and creates a suitable cellular microenvironment for virus replication (Table 1). Besides direct interaction with many host proteins, the AC2/C2 plays a key role in the geminiviral pathogenesis and involved in the transactivation of virion-sense promoter [8, 12]. All TrAP or AC2/C2 of begomoviruses contain a C-terminal acidic activation domain necessary for the transactivation of viral as well as host genes. The transcriptional activation property of C-terminal 30 amino acids (residues from 100 to 129) and a minimal activation domain (Residues from 115 to 129) of Tomato golden mosaic virus (TGMV) AC2 was previously confirmed using insect and yeast cell transfection experiments [13].The presence of transactivation domain in begomoviral AC2/C2 is essential for suppression of RNA silencing and blocking the spread of silencing signal [9]. The deletion of transactivation domain results in the depletion of transactivation property [8].

Though there are many experimental evidences for AC2/C2- mediated viral gene transactivation, the possible mechanism of transactivation was established by Ruiz-Medrano et al, (1999) via experiments with Conserved Late Elements (CLE, consensus GTGGTCCC). The transient expression of GUS constructs containing double copies of CLE motif in CaMV promoter with and without the Pepper huasteco virus (PHV), a member of bipartite begomovirus, was clearly established the importance of CLE in AC2-mediated transactivation [8]. Further, it is identified that a single repeat of CLE element can enhance the reporter luciferase gene expression up to two folds and multiple repeats of CLE can enhance the reporter gene expression up to fifteen folds. In addition to viral genes, AC2 transactivates a number of host genes upon infection. Upregulation of more than 160 host genes upon transient expression of Mungbean yellow mosaic virus (MYMV) and African cassava mosaic virus (ACMV) AC2 constructs in Arabidopsis protoplasts had clearly proved the transactivation property of AC2 [9]. The C-terminal region of begomoviral AC2/C2 comprises the acidic transcriptional activation domain which can also function in yeast and mammalian cells [13]. Hence, in the present study, a brief sequence analysis for the C-terminal region of begomoviralAC2/C2 protein has been done. Around 122 Arabidopsis thaliana promoters were mapped for having exact CLE motif (consensus - GTGGTCCC) in their promoter sequence and their corresponding genes were identified. These 122 CLE containing genes have more possibility for transactivation either directly or indirectly or repression by AC2 which may depend on the interacting partner of host protein. The interaction network of A. thaliana genes, those have CLE in their promoter sequence, are analyzed through STRING and PAIR web servers using their locus id. The interaction between the CLE containing protein and other cellular proteins were represented as colored nodal diagram and the functional details of each interacting proteins were also recorded. Protein interaction network analysis of 87 AC2 transactivated genes and 10 AC2 interacting genes were also identified and their functional properties were documented.

## Methodology

The A. thaliana cis regulatory sequences and promoter database were downloaded from Agris (http://agrisknowledgebase. org/AtcisDB/) website. List of A. thaliana promoters containing exact CLE element (consensus- GTGGTCCC) were shortlisted by using Linux 'grep' commands. The corresponding gene details were identified by searching TAIR (The Arabidopsis Information Resource) (https://www.arabidopsis.org/) and Plant Genome and System Biology (PGSB) (http://pgsb.helmholtzmuenchen. de/plant/index.jsp) database. Protein multiple sequence analysis was done by using NCBI cobalt (https://www.ncbi.nlm.nih.gov/tools/cobalt/re_cobalt.cgi) and Jalview software. The protein interaction network analysis for individual CLE containing genes and other AC2 transactivated genes of A. thaliana were done using STRING (https://stringdb. org/) webserver. The interconnectivity of protein interaction network of AC2 transactivated genes were identified using Predicted Arabidopsis Interactome Resource (PAIR) web server (http://www.cls.zju.edu.cn/pair/home.pair). In all cases, the gene locus ID was used for the data retrieval from TAIR and PAIR web servers. Protein interaction network map visualization and analysis of interacting proteins were done using Cytoscape3.6.0 (http://www.cytoscape.org/release_notes_3_6_0.html). For visualizing protein interaction maps, the SIF (*.sif) files of the query proteins are downloaded from the PAIR web server and visualized in cytoscape software. Making color images of protein sequence alignments and residue pattern analysis was performed using Jalview. Secondary structure analysis of AC2/C2 proteins was carried out using YASPIN (http://www.ibi.vu.nl/programs/yaspinwww/) and I-TASSER web server tools by uploading the fasta files containing begomoviral AC2/C2 protein sequences. Details of different begomoviral isolates were identified using ICTV (International Committee on Taxonomy of Viruses) begomovirus webpage (https://talk.ictvonline.org/ictvreports/ ictv_online_report/ssdnaviruses/ w/geminiviridae/392/genus-begomovirus) and the sequence details of different begomoviralAC2/C2 proteins were taken via protein sequence retrieval from NCBI protein webserver.

## Results

### Sequence analysis of C-terminal region of begomoviral AC2/C2

**Figure 1:**
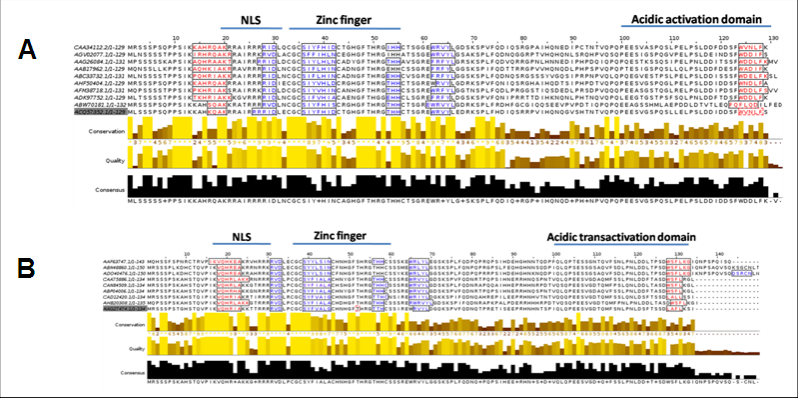
Multiple sequence alignment of begomoviral AC2/C2 protein. Multiple sequence alignment of Bipartite (A) and monopartite (B) geminiviral AC2/C2 proteins sequences shows helix forming residues (Red alphabets) and sheet forming residues (Blue alphabets). CAA34112.2 - AC2 of Abutilon mosaic virus, AGV02077 - AC2 of Cotton chlorotic spot virus AC2, AAG26084 - AC2 of Cucurbit leaf crumple virus, AAB17962 - AC2 of Cabbage leaf curl virus, ABC33732 - AC2 of Euphorbia mosaic virus, AHF50404 - AC2 of Jatropha mosaic virus, AFM38718 - AC2 of Jacquemontia mosaic Yucatan virus, ADK97752 - AC2 of Melon chlorotic mosaic virus, ABW70181 - AC2 of Macroptilium golden mosaic virus, ACQ57352 - AC2 Malvastrum yellow mosaic Jamaica virus, AAF63747 - C2 of Bhendi yellow vein mosaic virus, ABN48860 - C2 of Mesta yellow vein mosaic virus, ADO40476 - C2 of Okra enation leaf curl virus, CAA75886 - C2 of Papaya leaf curl virus, CAN84509 - C2 of Pedilanthus leaf curl virus, ABP04006 - C2 of Radish leaf curl virus, CAD12420 - C2 of Squash leaf curl Yunnan virus, AHB20308 - C2 of Tomato leaf curl virus, AAG2747.4 - C2 of Tomato yellow leaf curl China virus.

In the present study, a brief protein sequence analysis of Cterminal region comprising the acidic transactivation domain was carried out for various begomoviralAC2/C2 proteins. In order to understand and study the AC2/C2 in detailed manner, various isolates of begomoviruses were identified from ICTV website (https://talk.ictvonline.org/ictvreports/ ictv_online_report/ssdnaviruses/ w/geminiviridae/392/genus-begomovirus) and the begomoviralAC2/C2 protein sequences of different geographical isolates were downloaded from NCBI protein server (https://www.ncbi.nlm.nih.gov/protein/) for multiple sequence alignment studies. The secondary structure analysis of AC2/C2 reveals the presence of distinct alpha helix forming residues on both the N-terminal and C-terminal region. There are distinct patches of sheet forming residues situated in between residue 16 to 70of AC2 (Figure 1). The first half of the protein contains more sheet forming residues and similar kinds of residues are almost absent in the C-terminal region. When compared to the Nterminal region, the residues in the C-terminal end are less conserved. However, the position of various acidic, aromatic and hydrophobic residues remains conserved at the C-terminal region. In contrast, further deep analysis on the N-terminal end of AC2/C2 has revealed more of basic and other conserved cysteine residues, which are opposite to the C-terminal region bearing more number of hydrophobic residues.

The sequence analysis of bipartite AC2 protein reveals varying protein length from minimum of 128 (YP_001960954.1) to a maximum of 174 amino acids (NP_612596.1). In general, the begomoviral AC2/C2 proteins contain three structural domains such as the N-terminal arginine rich NLS, the middle zinc finger forming domain and the C-terminal acidic transcriptional activation domain. The C-terminal acidic transactivation domain is essential for viral gene transactivation. Hartits et al. previously identified the presence of 15 amino acid minimal transactivation domain of TGMV AC2, in 1999. The comparative analysis of TGMV 15 amino acid minimal transcription activation domain (SMDDIDDSFWENLFK) with 124 bipartite AC2 proteins had clearly showed the presence of difference in aminoacid composition. Analysis of residue identity between TGMV minimal transactivation domain and various bipartite AC2 proteins indicates difference in amino acid identity from 87 to 46%. Hence, residues from 100 to the end of protein were chosen for the sequence analysis of C-terminal region comprising acidic transactivation domain. The multiple sequence alignment of bipartite AC2 C-terminal region (residues from 100 to 174) indicates the presence of conserved acidic, aromatic and hydrophobic residues (Figure S1, S2, S3, S6, S7 and S8 - available with authors). Out of 124 bipartite AC2 proteins analyzed, ~84 proteins from different bipartite begomoviral isolates have conserved aromatic residues which form a pattern "FWXXXF" at the C-terminal region (Figure S1 - available with authors) (where F is Phenylalanine, W is Tryptophan and X represents any aminoacid). In addition to the previous pattern, the aromatic residues also form F-X3-FW-X3-F and F-X9-FW-X3-F patterns in the C-terminal region of bipartite begomoviral AC2 (Figure S1 - available with authors). The aromatic residues are found abundant between residues 35 to 90 of AC2 and are lesser in the C-terminal region. The conserved acidic residues forms distinct patterns AA-X9-A-X4-AAXAA-X4-A, AA-X14-AA-X2-A-X3-AA and A-X14-AAXAA-X4-A in the C-terminal region of AC2 (Where A represents any acidic residue and X-represents any amino acid) (Figure S2 - available with authors). The hydrophobic residues are spread throughout the protein but much more hydrophobic residues are localized in the zinc finger forming region. The conserved hydrophobic residues form three types of residue patterns such as 'H-X5--H-X2-H-X2-H-X2-H-X3-H-X3-HH', 'H-X7-H-X4-HH-X2-HX2- H-X2-HXH-X4-HH-X2-H'and 'H-X5-HHXH-X2-HXXH-X3- HXHXHH' in the C-terminal end of AC2 (Where H represents any hydrophobic residue and X represents any amino acid) (Figure S3 - available with authors). Apart from these AC2 proteins having easily recognizable patterns, few AC2 proteins do not have any specific patterns of acidic, aromatic and hydrophobic residues and these residues are found spread randomly in the C-terminus.

Similar kind of analysis on monopartite C2 proteins had revealed the presence of easily recognizable residue patterns with conserved acidic, hydrophobic and aromatic amino acids in the C-terminus (Figure S1, S4, S5, S9, S10 - available with authors). The length of the monopartiteC2 protein ranges from 128 (AAM74489.1) to 172 (YP_001876451.1) residues. Comparative analysis of 15 amino acid TGMV minimal transactivation domain (SMDDIDDSFWENLFK) with 463 monopartite C2 proteins using multiple sequence analysis had revealed differences in amino acid composition. Analysis of residue identity between TGMV minimal transactivation domain and various monopartite C2 proteins indicates difference in amino acid identity from 80 to 36%. Hence, in the present study, residues from 100 to end of the protein were chosen for the analysis of C-terminal region comprising acidic transactivation domain. Analysis of secondary structure of monopartite C2 proteins had revealed the presence of conserved α-helix forming residues on both the amino and carboxyl terminal regionsas similar to bipartite AC2 proteins (Figure 1). There are 3 to 4 patches of sheet forming residues are located in the N-terminal half of the protein (residues from 30 to 70) but similar patches are absent in the C-terminal half of the protein (Figure 1).

Based on the protein length, the monopartite C2 proteins can be divided into four groups for the convenience to group different residue patterns among various isolates. i.e Group A (contains 128 to 131 residues), Group B (contains 134 to 136 residues), Group C (contains 147 to 149 residues) and Group D (contains 150 to 152 residues).The proteins belong to each group have unique residue patterns based on the position of acidic, aromatic and hydrophobic amino acids in the C-terminal region. In most of the C2 proteins, the positions of aromatic residues were conserved and the C-terminal region of C2 (from 100th residue onwards) had lower number of aromatic residues when compared to the N-terminal region. Out of 463 C2 proteins analyzed, C2 protein from Honey suckle yellow vein mosaic virus (Protein id: BAD23955, BAF64256, BAF64262) had showed the presence of only one aromatic residue in its C-terminal region. Most of the aromatic residues were spread in between residues 35 to 90 of C2 and the aromatic residues of group A proteins form a distinct 'FWXXXF' pattern in the C-terminal end as similar to bipartite AC2.The Group B and D have distinct 'F X13-WXF' pattern in the C-terminal end (Where F and W represents phenylalanine and tryptophan respectively and X represents any amino acid). The group C mainly comprises the viral isolates of Sweet potato leaf curl virus which forms distinct 'WC-Y/F-SQLDWYF' pattern in the C-terminal region where the positions of aromatic residues are found to be conserved (Figure S1 - available with authors) (Y/F - either Y or F in the specific position) (Y represents tyrosine and F represents phenylalanine). The analysis of acidic residues in the C-terminal region of monopartite C2 indicates the presence of easily recognizable residue patterns. The group A monopartite C2 proteins have 'AX2- A-X11-AA-X6-AA', 'A-X14-AA-X2-A-X3-AA' and 'A-X9-A-X4- AA-X-AA-X4-A' patterns in the C-terminal. Group B proteins contain 'AA-X3-A-X10-AA-X4-A' and 'A-X3-A-X10-AA-X4-A' patterns in the C-terminal region. Group C proteins have 'A-X7- A-X6-A-X6-A-X5-AXAA-X9-A' and 'A-X11-AXA-X5-AA-X11-A-X6- A' patterns in the C-terminal region. The D group contain 'A-X14- AA-X4-A' pattern in the C-terminal end (where A represents any acidic residue and X represents any aminoacid) (Figure S4 - available with authors). About 99% of the monopartite C2 proteins have acidic amino acids in conserved positions on the Cterminal region and some of the C2 proteins do not have any acidic residue patterns as stated above and in those C2 proteins, the acidic residues are found to spread randomly in the Cterminal end. Generally the acidic amino acids in the transcriptional activation domain are necessary for the initiation of transacriptional activation. But there is a unique TrAP from Cyamopsistetragonoloba leaf curl virus (ADD62431.1), which does not have any acidic amino acid in its C-terminal end (from residue 100 to 148). There are two more monopartite C2 proteins from Tomato bright yellow mosaic virus (AGN12887) (from residue 100 to 152) and Tomato bright yellow mottle virus (AGN12891) (from residue 100 to 148)) which have only one acidic amino acid in the C-terminal region. The analysis of hydrophobic residues in the C-terminal region shows their arrangement in five different patterns. The group A proteins have 'H-X5-H-X2-H-X2-H-X2-H-X3-H-X3-HH' pattern. The B group proteins have 'HXH-X5-H-X4-HH-X2-H-X2-H-X2-H-X4-HHHH-X2- H' and 'H-X7-H-X4-HH-X2-H-X2-H-X2-HXH-X4-HH-X2-H' pattern. The C group proteins have 'HXH-X3-H--X4-H-X2-H-X3- HXXHXXH-X6-H-X3-H-X3-H' pattern. The D group proteins have 'HXH-X8-HXHHXXHXXHXXH-X6-HHXXH-X6-H-X5- HXH' pattern (where H represents any hydrophobic residue and X represents any amino acid) (Figure S5 - available with authors). Thus the positions of acidic, aromatic and hydrophobic amino acids differ with various begomoviral AC2/C2 proteins.

### Mapping Arabidopsis promoters having CLE motif

The report of rightward viral gene transactivation by AC2 via CLE elements has given a major clue for the possible transactivation of host plant genes, which harbor the same consensus CLE motif in their promoters. In order to understand whether host gene promoters have CLE, we analyzed Arabidopsis thaliana promoter database and have shortlisted 122 promoters for having exact CLE sequence consensus "GTGGTCCC" (Figure S12 - available with authors). Among the identified promoters and their corresponding genes, few were experimentally proved for their transactivation by AC2 [9, 14] (Table 2). Most of the identified proteins were transcription factors belong to bHLH, MYB and other DNA binding proteins suggesting that AC2 might transactivate these proteins to evade specific cellular processes critical for viral survival. The identified genes having CLE in their promoter includes genes from nucleus, cytoplasm, chloroplast and other membrane structures. Many of them are involved in plant defense processes and some are subunits of large complex proteins. In addition, some proteins were reported for their involvement in basic transcription, chromatin remodeling and plant defense process.

### Protein interaction network analysis

It is well known that AC2/C2 directly interacts with many host proteins and modulates the host gene expression through trans activation property. But the mechanism behind the regulation of host proteins for AC2-mediated pathogenesis, transactivation and neutralizing host defense remains elusive. Analysis of protein interaction networks involving AC2/C2 and the host proteins might give a better understanding on how AC2/C2 regulates different host cellular proteins for viral pathogenesis. The interaction between the AC2/C2 and the key proteins of plant metabolism and other host proteins, those have either direct or indirect interaction with the AC2/C2 interacting proteins, could reveal the regulation of much more host proteins. There are many research articles having experimental data for the host gene transactivation where a number of host genes are transactivated upon AC2 infection [15]. Hence, in the present study, a number of AC2 trans activated genes from A. thaliana were selected for protein interaction network studies. The up regulation of ~163 A. thaliana genes upon AC2 infection was experimentally proved by Trinks et al. in 2005. Out of the163 genes, they have confirmed the definite upregulation of 90 genes by applying most stringent criteria and cross comparison between biological replicates [9]. These 90 genes are either trans activated by both MYMV and ACMV AC2 or at least by one among the two TrAP. Hence, these 90 A. thaliana genes were selected for protein interaction network analysis via STRING web-server. Out of 90 genes, 87 genes were found to have extensive protein interaction networks with various A. thaliana proteins (Figure S15 - available with authors). Analyzing all kinds of protein-protein interactions involving these 87 transactivated genes via PAIR web server had clearly showed the involvement of more than 890 Arabidopsis proteins having 1000 number of interaction with AC2 transactivated genes either directly or indirectly. This indicated the interconnection between the trans activated genes and the possibility of coordination between number of host genes in AC2- mediated pathogenesis (Figure S16 & S17 - available with author). Further many experimental evidences were previously published for the direct interaction between the Gemini viral proteins and the host plant proteins (Table 1). These interactions are important which lead to successful viral pathogenesis either directly or indirectly. Thus, in the present study, the interaction network analysis of A. thaliana proteins, those have direct interaction with begomoviral AC2 (i.e. SNF1 kinase, SAMDC1, Adenosine kinase, rgsCaM, AtAGO1 and signallosome proteins), is carried out. The results had revealed the possible involvement of ~120 more Arabidopsis thaliana proteins in AC2-mediated pathogenesis (Figure S14 - available with authors). The functional analysis of each protein had showed their role in diverse cellular processes.

**Figure 2:**
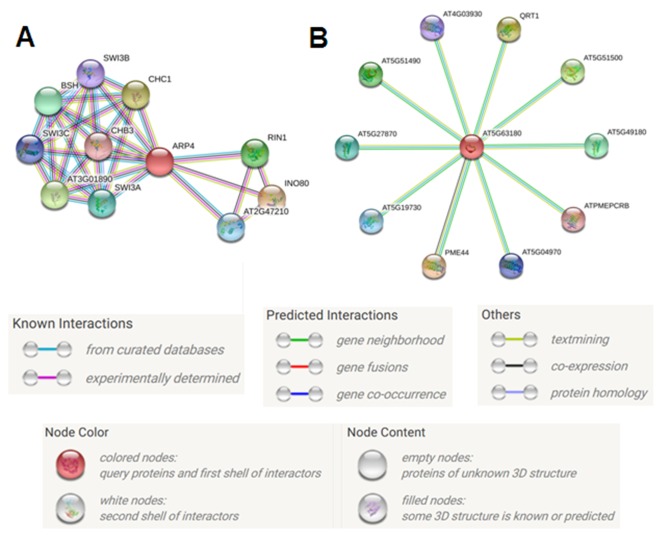
Protein-Protein interaction network analysis of proteins having CLE in the promoter sequences. Protein interaction network of (A) Arabidopsis thaliana Actin related protein 4 (ARP4 - AT1G18450.1) and (B) Arabidopsis thaliana pectin lyase-like super family protein (AT5G63180.1). SWI3B - Switch subunit 3; Component of a multiprotein complex equivalent of the SWI/SNF complex, BSH - BUSHY GROWTH protein, SWI8C - SWI/SNF complex subunit SWI3A, AT3G01890 - SWIB/MDM2 domain super family protein, SWI3A - SWI/SNF complex subunit SWI3A, CHB3 - SWI/SNF complex subunit SWI3D, CHC1 - SWI/SNF complex component SNF12-like protein, RIN1- RuvB-like protein 1, AT2G47210 - DNA methyltransferase 1-associated protein 1, INO80 - DNA helicase and probable main scaffold component of the INO80 complex, SWI3C - SWI/SNF complex subunit SWI3C, AT4G03930 - , QRT1 - QUARTET 1; Pectinesterase protein, AT5G51500 - Putative pectinesterase/pectinesterase inhibitor 60, AT5G49180 - Putative pectinesterase/pectinesterase inhibitor 58, ATPMEPCRB - Pectinesterase 41, AT5G04970 - Putative pectinesterase/pectinesterase inhibitor 47, PME44 - Pectinesterase 44; Acts in the modification of cell walls via demethylesterification of cell wall pectin, AT5G19730 - Pectinesterase; Acts in the modification of cell walls via demethylesterification of cell wall pectin, AT5G27870 - Pectinesterase 28; Acts in the modification of cell walls via demethyl esterification of cell wall pectin, AT5G51490 - Putative pectin esterase/pectin esterase inhibitor 59.

Previous studies with the CLE consensus "GTGGTCCC" in A. thaliana genome have led to the identification of 122 genes for having exact CLE motif in their promoter sequences. These genes have high possibility to get trans-activated by AC2 and thus they are chosen for the protein interaction network analysis via STRING server. Among the 122 identified genes, around 106 genes had showed different interaction networks with a wide variety of cellular proteins (Figure S13 - available with authors). Analysis of their functional details indicates their involvement in diverse cellular processes such as cell wall modification, dehydration tolerance, transcriptional regulation, nucleic acid methylation, cell cycle regulation, leaf senescence, signal response and chlorophyll synthesis. In the interactome diagram, the proteins, which contain the CLE in their promoter, were shown in red color circle, and the other interacting proteins were shown with variously colored circles (Figure 2). Each circle represents a protein with various colors, which are connected with other proteins and eventually form a network of protein interaction. It is interesting to underline that most of the interactions in the network is confirmed by experimental evidences such as co-immunoprecipitation, protein homology, text mining and data from curetted databases [16]. The identity and functional characteristics of interacting proteins are identified with the same web-server and tabulated along with the interaction map. Through this interaction network analysis, the ability of begomoviral AC2/C2 to influence many plant cellular processes becomes visible and the interaction networks themselves show how multiple proteins are regulated forAC2/C2 mediated pathogenesis.

## Discussion

Plants employ different kinds of defense pathways like mRNA silencing at post-transcriptional level (PTGS) and transcript silencing at transcriptional level (TGS) by DNA methylation to repress the protein synthesis of invading pathogens. To counteract these effects, plant viruses encode silencing suppressor proteins such as AC1, AC2/C2, AC4/C4, V2 and C1 of beta satellite which interact with a wide variety of host proteins and neutralize the host defense efficiently [17, 18]. Among the geminiviral suppressor proteins, the begomoviralAC2/C2 is a multifunctional protein which either trans-activates or inactivates key host proteins for the viral survival in the cell [8, 12] (Table 2 and 3). All the begomoviral AC2/C2 proteins have acidic transactivation domain in the Cterminal region. The presence of minimal activation sequences in the C-terminal region has been identified in TGMV AC2 by Hartitz et al. in 1999 [13]. The acidic activation domain is important for proper functioning of AC2/C2 and the property of host and viral gene transactivation has been lost in the absence of acidic transactivation domain [9]. Therefore, to understand the transactivation domain properties of begomoviral AC2/C2, we performed a protein sequence analysis on the C-terminal region of AC2/C2. Comparative analysis of the C-terminal 15 amino acid long (residues 115 to 129) minimal activation domain sequence (SMDDIDDSFWENLFK) of TGMV AC2 with different begomoviral AC2/C2 protein sequences was carried out by multiple sequence alignment. This comparative analysis showed significant difference in amino acid composition and the percentage of identity from AC2/C2 protein sequences that align with the minimal activation domain of TGMV AC2. Hence composition and positions of amino acids in the minimal activation domain sequence vary with the AC2/C2 of various begomoviral isolates. Analysis of secondary structure of begomoviral AC2/C2 proteins showed the presence of distinct helix forming residues on both C-terminal and N-terminal region. Generally the transcription activator proteins have DNA binding domain and transcription activation domain where the DNA binding domain bind to the DNA at specific locus and the transactivation domain interacts with various co-activators, mediators and components of transcription machinery during the process of transcriptional activation. The presence of helix in the transcriptional activation domain is advantageous since it enhances the interaction between the transcriptional machinery proteins and other cofactors. The transcriptional activation domain of certain transcription factors form α-helix during interaction with the transcriptional machinery or other cofactors [19, 20, 21]. The sheet forming residues are exclusively present in the N-terminal region of AC2/C2, which is completely absent in the C-terminal region. Hence, the N-terminal with zinc finger domain and beta sheets can bind the DNA and the C-terminal region with α-helix could do transcriptional activation process. Compared to different classes of transcriptional activation domains, (i.e. acidic, Proline rich, Isoleucine rich and Glutamine rich transcriptional activation domains) the acidic transactivation domain is a widespread domain among the transcription activator proteins. The position and number of available acidic, aromatic and hydrophobic residues are important factors determining the activity of acidic transcriptional activator domains. Hence, an analysis on acidic, aromatic and hydrophobic residues in the C-terminal region of AC2/C2 was performed. The role of acidic amino acids in the induction of transcriptional activation has been proved with many transcriptional activator proteins [22]. The essential interaction between the hydrophobic residues with the proteins of transcriptional machinery and the direct correlation of hydrophobic residues with the strength of activation domain was well established with many transcriptional activator proteins [23, 24]. Similarly, enhancement of transcriptional activation was observed with increase in aromatic residues was demonstrated with some transcriptional activator proteins [25]. Additionally, a significant reduction of transactivation property was observed when the aromatic residues of acidic transactivation domain of TrAP was substituted with non aromatic residues [19]. Hence, the analysis of acidic, aromatic and hydrophobic residues in the transactivation domain is important and the analysis of begomoviral AC2/C2 proteins of different geographic regions has revealed the presence of average of 6 to7 acidic amino acids in the C-terminal end (from residues 100 to 130). In many begomoviral AC2/C2 proteins, the positions of the acidic and hydrophobic residues were found conserved in the C-terminal region and form easily recognizable patterns (Figure S2 & S4 - available with authors). However, when compared to typical acidic transcriptional activation domain, the presence of triple acidic residues (DDD, EEE) are extremely rare in the transactivation domain of begomoviral AC2/C2 and the acidic residues are randomly distributed either as singles orin pairs. The triple acidic residues in the C-terminal region was rarely seen with two monopartite C2 proteins such as the C2 of Horsegram yellow mosaic virus (protein id: NP_981937) and Pepper huestaco yellow vein virus (Protein id: NP_040323.1). Out of 463 monopartite C2 proteins analyzed, one C2 protein (Protein id: ADD62431) was found for not having any acidic amino acids in the C-terminal end (from residue 100 to 148) and two C2 proteins (Protein id: AGN12887 and AGN12887) were found for having only one acidic residue in the C-terminal end (From residue 100 to 152). Hence, experimental studies are needed for the identification of residues essential for the initiation of transcriptional activation in these proteins. Similar observation on aromatic residues in the C-terminal region of monopartite C2 of Honey suckle yellow vein mosaic virus (Protein id: BAD23955, BAF64256, BAF64262) had revealed the presence of only one aromatic residue (from residue 100 to 135). Thus, experimental studies are needed to establish the role of C-terminal aromatic residues in transcriptional activation. Aligning begomoviral AC2/C2 proteins on the basis of protein length had showed the presence of easily recognizable patterns of acidic, aromatic and hydrophobic residues in the C-terminal region. Based on the analysis, there are 3 acidic, 3 aromatic and 3 hydrophobic residue patterns identified in the C-terminal region of bipartite begomoviral AC2 proteins. There are about 8 acidic, 3 aromatic and 5 hydrophobic residue patterns identified in the C-terminal region of monopartite begomoviral C2 proteins (Figure S1, S2, S3, S4 and S5 - available with authors). Thus, the positions of acidic, aromatic and hydrophobic residues of C-terminal region vary between different isolates of begomoviral AC2/C2 and forms easily recognizable patterns. The residue distribution analysis had clearly revealed the presence of maximum number of aromatic residues in between residues 35 to 95 of AC2/C2 (Figure S6 & S9 - available with authors). The hydrophobic aminoacids are spread throughout the protein and their positions in the AC2/C2 found to be conserved (Figure S8 & S11 - available with authors). Even though the hydrophobic residues spread all over the protein, they were clustered near the conserved cystein residues. These observations are in line with other transcriptional regulator proteins since the zinc finger and hydrophobic residues are necessary for proper folding and functioning of proteins. The protein interactome analysis of 87 MYMV and ACMV AC2 transactivated Arabidopsis genes via STRING and PAIR web servers had showed the possible regulation of more than 890 host proteins with 1000 number of protein interactions (Figure S15, S16, S17 - available with authors). Further, it becomes clear that the identified interacting genes form a huge interaction network by having extensive interactions between themselves (Figure S16 - available with authors).

Similar studies with Arabidopsis thaliana proteins, those have direct interaction with AC2/C2 (SNF1, SAMDC1, ADK, rgsCaM, AtAGO1 and signallosome proteins), also indicate the possible involvement of ~120 more host proteins in AC2-mediated pathogenesis (Figure S14 - available with authorss). SNF1 is a kind of serine/threonine kinase protein, which plays a central role in the regulation of metabolism. Inactivation of SNF1 by geminiviral AL2 and L2 proteins was well established [26] and the protein interaction network of SNF1 had revealed the interaction of 10 other host proteins. In the same way, SAMDC1 (Sadenosylmethioninedecarboxylase 1) is an essential enzyme in polyamine synthesis, needed for the conversion of SAM to decarboxylated S-adenosylmethionine (dcSAM). Both SAM and dcSAM were needed for many metabolic pathways including maintenance of methylation cycle, spermineand spermidinebiosynthesis, transmethylation, ethylene biosynthesis, and trans-sulfuration. The attenuation of SAMDC1 degradation by BSCTV C2 was well established and this process helps the virus to escape from DNA methylation mediated gene silencing [27]. Similarly, suppression of DNA methylation by C2 of Bhendi Yellow vein mosaic virus (BYVMV) was well established through quantitative PCR (qPCR) and bisulfite-next generation sequencing (NGS) methods [29]. Analysis of protein interaction network of SAMDC1 had revealed the interactions of 10 more host proteins (Figure S14 - available with authorss). The interaction and inhibition of Adenosine kinase (ADK) by geminiviral AL2 and L2 was proved using yeast cells and transgenic plants expressing AL2 and L2 [28]. The analysis of protein interaction network of ADK had revealed the possible regulation of 9 different host proteins (Figure S14 - available with authors).

Further observation of CLE consensus (GTGGTCCC) on the Arabidopsis gene promoter, we discovered 122 promoters for having exact CLE motif. Among 122 genes having CLE, a few of them were significant in relation to plant metabolism such as Actin related protein 4 (ARP4), Proteins those having bHLH domain, F-box proteins, Auxin related proteins, Kinase family proteins, MYB domain containing proteins, DNA unwinding and many transcription factor proteins. The array of functions from the identified genes containing CLE in their promoter indicates that AC2/C2 can transactivate a wide variety of host genes, those functions in most of the cellular processes. As presented in the list, a set of similar kind of gene upregulation was proved upon AC2 infection by various researchers [9, 14]. It should be noted that all the computationally predicted 122 genes having exact CLE were not experimentally proved for their upregulation either by MYMV or ACMV or PHV AC2. The computational analysis shows the possibility for the transactivation of 122 CLE containing gene by PHV AC2 since they have exact CLE consensus "GTGGTCCC" in their promoter sequences. But upregulation of predicted CLE containing genes by the PHV AC2 are needed to be proved by a series of experiments. Generally the upregulation of transcripts upon AC2 infection should be due to direct or indirect effect of AC2 and its interacting partners from the host, which may determine the fate of gene transcription. However, this hypothesis for the curtailed gene expression should be investigated by designing proper methodology. Out of 122 identified CLE containing genes, about 106 genes had showed extensive protein interaction network (Figure S13 - available with authors). The analysis of protein interaction networks of identified 106 CLE containing genes had showed the involvement of much more cellular proteins in AC2-mediated viral pathogenesis. Generally, innumerable protein-protein interactions were happens in a living cell and the investigation of protein interaction network is much more helpful to increase the discovery power of noisy data, interpreting the genome wide association of genes and to identify new molecular players. The analysis of interacting proteins on each protein interaction networks ofAC2 transactivated, AC2 interacting and CLE containing A. thaliana genes indicates the possible regulation of hundreds of cellular proteins by begomoviral AC2. However this needs to be confirmed by series of experiments. Though begomoviruses encode few proteins in their genome, they can utilize the host machinery by interaction with key cellular proteins and regulating necessary proteins by exploiting extensive protein interaction networks.

## Conclusion

Detailed analysis on the C-terminal region of various begomoviral AC2/C2 proteins revealed the presence of major differences in amino acid composition and position of acidic, aromatic and hydrophobic residues. Mapping of Conserved Late Element (CLE consensus-GTGGTCCC) in A. thaliana promoters had led to the identification of 122 promoters and their corresponding genes for the possible transactivation by PHV AC2.The protein interaction network of CLE containing, AC2 interacting and AC2 transactivated A. thaliana genes show the possible regulation of hundreds of A. thaliana proteins by begomoviralAC2. Since the begomoviruses encode limited proteins in their genome, the extensive exploitation of host protein interaction networks by AC2 helps begomoviruses to regulate much more host proteins to establish a successful infection.

## Figures and Tables

**Table 1:** List of host proteins those interacting directly with Geminiviral AC2/C2 protein

S. No	Geminiviral AC2/C2 protein	Name of the host protein	Notes	References
1	CaLCuV and SCTV AC2/C2	SnRK1.2	SnRK1.2 shows strong interaction with AC2/C2 and silencing of SnRK1.2 makes the plant more susceptible to the virus.	[15]
2	MYMIV-AC2	AGO1	MYMIV-AC2 interacts with AGO1 and inhibits transcript silencing activity invitro.	[30]
3	MYMIV-AC2	RDR6	MYMIV-AC2 interacts with RDR6 and interferes in its activity.	[30]
4	TGMV - AL2 and BCTV - L2	SNF1	AL2/L2 inactivates SNF1 directly. Silencing SNF1 makes the plant more susceptible and High expression of SNF1, increases plant resistance to geminiviruses.	[26]
5	BCTV-L2	Adenosine kinase	BCTV-L2 interacts and inactivates Adenosine kinase - a nucleoside kinase that catalyzes the salvage synthesis of 5'-AMP from adenosine and ATP	[28]
6	BSCTV-C2	SAMDC1 (S- adenosyl-methionine decarboxylase 1)	BSCTV-C2 attenuates the 26S-proteasome mediated degradation of SAMDC1	[27]

**Table 2:** List of Arabidopsis thaliana promoters having CLE sequence "GTGGTCCC" with their corresponding genes and upregulation of similar transcripts.

S.No	List of few Arabidopsis thaliana promoters having exact 'CLE' consensus "GTGGTCCC"	Name of the corresponding gene	Cellular localization	List of similar transcripts up regulated by AC2 - proved by experimental methods in Arabidopsis thaliana
1	At1g18450.1	Actin Related Protein 4	Nucleus	Two fold increase of transcript level was confirmed by semi quantitative reverse transcription PCR [14]
2	At2g38090.1	MYB Family transcription factor	Nucleus	Transcript (MYB Family transcription factor proteins - AT1G70000, AT2G38090, AT1G74430, AT1G22640) upregulation confirmed upon geminivirus AC2 infection by CHIP based transcriptome sequencing [9].
	At5g26660.1			
3	AT1G66470	BHLH DNA binding super family Protein	Nucleus	Transcript (BHLH DNA binding super family protein - AT1G66470) upregulation confirmed upon geminivirus AC2 infection by CHIP based transcriptome sequencing [9].
	At1g10585.1			
	At3g25710.1			
	At4g09820.1			
	At4g36930.1			
	At5g08130.1			
	At5g50915.1			
4	At5g52320.1	Cytochrome P450	Plant microsomes and endoplasmic reticulam.	Transcripts (Cytochrome P450 family proteins - AT5G25120, AT3G03470) upregulation confirmed upon geminivirus AC2 infection by CHIP based transcriptome sequencing [9].
	At2g45580.1			
	At3g26320.1			
5	At4g34440.1	Serine/Threonine protein kinase	Chloroplast	Serine/Threonine protein kinase (AT5G63650, AT5G08590, AT3G46280) upregulation has been confirmed with ACMV and MYMV AC2 infection by CHIP based transcriptome sequencing [9].
	At1g53165.1			
6	At1g64000.1	WRKY family transcription factor	Nucleus	Transcript (WRKY family transcription factor protein - AT4G01250 - ATWRKY22 (It is involved in regulation of dark induced leaf senescence)) upregulation confirmed upon geminivirus AC2 infection by CHIP based transcriptome sequencing [9].
7	At5g40050.1	F-box family protein	Nucleus	F-box protein (AT1G78100) upregulation has been confirmed with ACMV and MYMV AC2 infection by CHIP based transcriptome sequencing [9].
	At1g57790.1			
	At3g62980.1			
	At4g35930.1			
8	At5g17030.1	UDP glucose: flavonoid 3-o-glucosyltransferase like protein	Cytoplasm	Transcript (UDP glucose: flavonoid 3-o-glucosyltransferase like protein - AT5G17050) upregulation by AC2 was reported by CHIP based transcriptome sequencing [9].
9	At5g24210.1	Alpha/beta-Hydrolases super family protein.	Cytoplasm	Transcript (Alpha/beta-Hydrolases proteins - AT3G50440) upregulation by AC2 was reported by CHIP based transcriptome sequencing [9].
10	At5g24540.1	beta Glucosidase	Cytoplasm	Transcript (beta glucosidase - AT3G07320) upregulation by AC2 was reported by CHIP based transcriptome sequencing [9].

**Table 3:** List of Arabidopsis thaliana genes having CLE sequence "GTGGTCCC" in their promoters with experimental evidence for down regulation of similar kind of transcripts upon Gemini viral AC2 infection.

S.No	List of Arabidopsis Promoter containing Exact 'CLE' consensus 'GTGGTCCC'	Name of the gene	Cellular localization	List of similar transcripts those were down regulated by AC2 protein in Arabidopsis thaliana (Liu et al., 2014) [15]
1	AT1G54310	S-adenosyl-L-methionine-dependentmethyltransferases super family protein.	Nucleus	AT5G66180, AT5G37990
	At2g43910.1			
2	At3g62980.1	TIR1 (Transport inhibitor response 1); Auxin binding / Protein binding / Ubiquitin-protein ligase	Cytoplasm	AT3G62980
3	AT2G41080	Tetra trico peptide repeat (TPR)-like super family protein	Cytoplasm	AT1G17680, AT5G51340, AT1G31840, AT3G24000, AT3G51280, AT5G53080, AT4G04370, AT1G76630, AT1G55890
	At5g14770.1			
4	At1g64000.1	WRKY56 transcription factor	Nucleus	AT1G69310 (WRKY57), AT5G52830 (WRKY27), AT2G38470 (WRKY33), AT4G12020 (WRKY19)
5	At1g09450.1	Protein Kinase super family protein	Cytoplasm	AT5G37790, AT1G71810, AT1G65950
6	At5g24540.1	Beta glucosidase	Cytoplasm	AT5G28510, AT1G66280, AT4G00100, AT5G24550
7	At5g63180.1	Pectin-lyase super family protein	Cytoplasm	AT4G13760, AT4G33440, AT3G24130
8	At1g07210.1	Ribosomal protein S18	Cytoplasm	RPS18
